# Awareness of Menstrual Abnormality Amongst College Students in Urban Area of Ile-Ife, Osun State, Nigeria

**DOI:** 10.4103/0970-0218.62559

**Published:** 2010-01

**Authors:** OA Esimai, GO Omoniyi Esan

**Affiliations:** Department of Community Medicine, Obafemi Awolowo University ILE-IFE, Osun State, Nigeria; 1 Department of Morbid Anatomy, Obafemi Awolowo University ILE-IFE, Osun State, Nigeria

**Keywords:** Awareness, health seeking behavior, menstrual abnormalities

## Abstract

**Background:**

Disturbances of menstrual bleeding are major social and medical problem for women and account for high percentage of gynecological visit.

**Objectives:**

The objective of the study was to document menstrual abnormalities experienced by female college students, their awareness and health seeking behavior.

**Materials and Methods:**

A cross-sectional survey was undertaken, 400 students were selected using stratified sampling technique and interviewed using semi-structured self-administered questionnaire. Inferential statistical analysis such as Chi-square test and logistic regressions were carried out.

**Results:**

The mean age at menarche was 14.18 years. Irregular menstrual cycles were reported in 9.0%. Dysmenorrhea was present in 62.5%, and 12.5% reported school absenteeism. Students' awareness of menstrual abnormalities was poor (29%). A few of them (10.5%) decided to seek help for menstrual abnormalities. The awareness of students on menstrual abnormalities was significantly influenced by their age (OR = 2.33, *P* = 0.03); however, age at menarche and level of study did not influence their awareness (OR = 0.45, *P* = 0.24 and OR = 1.42, *P* = 0.12). History of dysmenorrheal (OR = 10.2, *P* = 0.001) and academic disturbance (OR = 5.45, *P* = 0.001) had significant influence on the health seeking behavior of the students.

**Conclusion:**

There was a general lack of information about menstrual issues and when to seek help. There is a need to educate female college students about menstrual issues in order to improve their health seeking behavior as regards menstrual abnormalities.

## Introduction

Menstrual problems are generally perceived as only minor health concerns and thus irrelevant to the public health agenda, particularly for women in developing countries who may face life-threatening conditions.(1) Menstruation is a normal physiological process that begins during adolescence and may be associated with various symptoms, occurring before or during the menstrual flow. The frequency of irregular cycles ranged from 5 to 16%, with higher prevalences generally reported after medical interviews (9 to 16%) than lay interviews (5 to 15%).(2) The normal menstrual cycle relies on action and interaction of hormone released from hypothalamus-pituitary and ovaries and their effect on the endometrium. The normal menstrual pattern is such that age of menarche is less than 16 years, length of menstrual cycle 24-32 days, length of flow 3-7 days and amount of flow ≤80mL. Common menstrual disorders include heavy flow (menorrhagia), unusually light (hypomenorrhea), unusually frequent (polymenorrhea), unusually infrequent (oligomenorrhoea) and unusually painful (dysmenorrhea). Women can experience a variety of menstrual disorders. The most prevalent menstrual disorders among adolescents are excessive uterine bleeding, dysmenorrhea and premenstrual syndrome. Dysmenorrhea, usually of the primary type, is a common symptom and a common cause of school absenteeism among adolescents.(3,4) Most studies on dysmenorrheal have focused on adolescents. A study conducted amongst Nigerian university students reported prevalence of 72%.(5) In the absence of appropriate pain relief, women with severe dysmenorrhea may not be able to carry out their normal activities.

Although it is normal physiological process, many adolescents have little or no information about normal and abnormal menstruation.(6) There is a lack of current information concerning the knowledge and attitudes of urban adolescents regarding menstruation,(4) with a report that twice as many African-American adolescents felt unprepared and did not receive information about menarche when compared with Caucasian teens.

A review carried out among boys and girls in Middlesex County by Governors Task force showed that information received in the health education classes do not include information on normal and abnormal menstrual cycle. Some of the girls complained of excessive menstrual flow but do not take specific action. They usually did not consider the school nurse as a resource and often used other medical excuses to leave school. Both boys and girls are interested in knowing more about normal and abnormal menstruation; the girls so that they could make correct decisions on when to seek medical attention and boys so that they could support their female friends.(7) However studies have shown that most of what they know is often information obtained from their mothers and their peers.(8) The report of a study conducted amongst rural adolescent in India showed dysmenorrhea and irregular menses as the commonest menstrual problems of which only 5.3% consulted a doctor and 22.4% took over counter medications.(9) There are only limited number of studies on the level of information and understanding of bleeding disorders in women; nevertheless, the studies have dearth of information in the medical community and lay public about normal vs. abnormal menstrual bleeding.(7) The literature suggests that menstrual problems may be as common in developing countries as they are in developed countries, and that when services are available, this will prompt women to seek care for menstrual complaints.(10)

This study was carried out to determine the menstrual abnormalities experienced by young female college students and their awareness and health seeking behavior. This information will be useful in modifying health promotion and education activities for young females in this environment with a view to improving reproductive health services.

## Materials and Methods

### Study location

Universal Tutorial College lle-lfe, Osun state Nigeria, is a private polytechnic college approved by the State Government. It has an estimated population of 15000 with male and female students (2:3) in two levels of National Diploma degree program (ND I and II), taking art and social science courses in four departments namely accounting, marketing, languages and literature.

### Sampling method

Multistage Sampling Technique. The school was stratified based on the departments. The female students were selected from the two levels (ND I and II) in the four departments using the random sampling technique, and to avoid overrepresentation of any of the levels in the four departments, the students were selected propionate to their estimated population. The estimated population of the female students in each department was obtained from the school records.

### Data collection

Data was collected using pre-tested self-administered questionnaire, background information on respondent such as age and marital status. Questions related to menstruation elucidated variations in menstrual patterns: Cycle length, duration of flow, amount of flow and pain with menstruation. Respondents were also asked whether they had consulted any physician on their menstrual problems. Further questions were asked to assess their level of awareness on menstrual abnormalities.

### Data analysis

SPSS version 11 was employed for the data entry process and analysis was done using STATA version 8. Quantitative data was subjected to univariate analysis and statistical test of association such as Chi-square and logistic regression analysis. The level of significance was determined at *P* < 0.05.

## Results

The age of female college students ranged between 20 and 27 years with a mean of 21.1 years. The mean age at menarche was 14.2 years with a range of 10-17 years. The mean cycle length was 29.9 days, the range was 20 to 36 days and 9% had irregular cycle. About 18 (4.5%) reported duration of flow of less than 2 days, 2 (0.5%) duration of flow of over 8 days and 4 (1.0%) reported heavy menstrual flow as shown in Table 1.

**Table 1:** Menstrual patterns of the respondents *N* = 400

Menstrual characteristics	Frequency	Percentage
Age at menarche (years)		
<10	18	4.5
11-13	166	41.5
14-16	192	48.0
>17	24	6.0
Duration of flow		
<2	18	4.5
2-4	248	62.0
5-7	132	33.0
>8	2	0.5
Amount of flow		
Little (≤4 pads/day)	382	95.5
Moderate (5-7 pads/day)	14	3.5
Heavy (≥8 pads/day)	4	1.0
Cycle length		
≤20 days	7	1.7
21-35	283	70.8
≥36	74	18.5
Irregular pattern	36	9.0
Painful menstrual flow (dysmennorhea)		
No	158	39.5
Yes	242	60.5

Dysmenorrhea was reported in 242 (60.5%) of the students and 50 (12.5%) of respondents reported that the pain interfered with their academic activities.

One hundred and sixteen (29.0%) were aware of menstrual abnormalities as problem and 43 (10.8%) decided to seek medical attention. The age of students significantly influenced the awareness of students on menstrual abnormalities (OR 2.33, *P* =0.03) -older age group were more aware of menstrual abnormalities as compared with the younger age group. Age at menarche (OR = 0.45, *P* = 0.24; OR = 0.46, *P* = 0.07) and level of study (OR = 1.42, *P* = 0.12) had no significant influence on the awareness of students on menstrual abnormalities [Table 2].

**Table 2:** Factors that influence students' awareness of menstrual abnormalities

Factors	Odds ratio	*P* value	95% CI
Age of respondents			
<20 (ref)	1.00		
20-24	1.69	0.08	0.95-3.02
≥25	2.33	0.03	1.07-5.09
Age at menarche			
≤10	0.45	0.24	0.12-1.68
11-16	0.46	0.07	0.20-1.06
≥16 (ref)	1.00		
Level			
ND I	1.42	0.12	0.91-2.20
ND II (ref)	1.00		

Ref = Reference group

History of dysmenorrheal (OR = 10.2, *P* = 0.001) and academic disturbance (OR = 5.45, *P* = 0.001) were the factors that influence the students decision to seek medical attention for menstrual abnormalities. Age of the respondents (OR = 1.69, *P* = 0.26; OR = 2.73, *P* = 0.08) had no significant influence on the students decision to seek medical attention [Table 3].

**Table 3:** Factors that influence students' health seeking behavior on menstrual abnormalities

Factors	Odds ratio	*P* value	95% CI
Age of respondents			
<20 (ref)	1.00		
20-24	1.69	0.26	0.68-4.21
≥25	2.73	0.08	0.89-8.41
History of dysmenorrhea			
No dysmenorrhea (ref)	1.00		
Dysmenorrhea	10.3	0.001	3.16-33.7
History of academic disturbance			
No (ref)	1.00		
Yes	5.45	0.001	2.68-11.1

Ref = Reference group

## Discussion

The mean age of the respondents was 21.1 years, and attaining age at menarche at 14.2 years was higher than what obtained in previous studies,(11) because most studies were carried out amongst young and old adolescents. The finding of 1.7% of students with length of cycle less than 21 days showed a much lower prevalence compared with 24% of adolescent females reported having cycles shorter than 21 days in Nigeria.(12) The finding of 1% of the student having excessive or heavy menstrual flow was lower than the prevalence of 4% of women with heavy or prolonged bleeding reported in a study carried out in Gambia.(2) The report of 5 to 17% women experiencing irregular cycles in a survey carried out in developing countries(1) was consistent with the report of irregular cycle occurring in 9% of the female students in this present study. The differences observed in the prevalence of menstrual abnormalities might be due to factors such as anthropometric factors, which were influenced by diet as a function of the present socioeconomic situation of the country.

As might be expected, many women experience pain during menstruation, such pain was reported in 60.5% of the young female college student, only 12.5% reported interference with academic activities. These finding was consistent with the report of 16-58% of adult women and 35-78% of adolescent experiencing pain during menstruation and about 3-20% reporting it as severe enough to interrupt daily activities.(1)

Awareness of the students on menstrual abnormalities was poor in the study; this was consistent with a report of poor awareness about the process of menstruation in a previous study carried out amongst rural adolescent girls.(9) The older students were more aware of abnormal menstruation in the present study, this was supported by the previous report of the older women with more education having better awareness of health issues and are more likely to seek medical attention including antenatal clinical attendance where they may receive information regarding issues surrounding the menstrual cycle.(13) However, the above report was not in agreement with the present finding of no significant association between the level of study of the respondents and their awareness on abnormal menstruation.

About 10.5% of the students decided to seek medical attention for menstrual abnormalities. This was far above the report of 5.3% rural young adolescent consulting doctors while 22.4% took over-the-counter medications in a previous study.(9) This finding was in keeping with the previous report of women not seeking medical attention for menstrual-related symptoms because menstruation is considered personal and highly secretive, or because of fear they prefer self medication as alternative technique.(2–14,15) Multivariate analysis indicated that students who had dysmenorrhea were more likely to seek medical care, and students who reported the pain severe enough to disturb their academics were also more likely to seek care than their peers. This was in keeping with previous report of severity of pain as a factor affecting decision to seek medical care for dysmenorrhea.(16) A previous study reported that the older age group with more education had better awareness of health issues and are more likely to seek medical attention including antenatal clinical attendance where they may receive information regarding issues surrounding the menstrual cycle. This was not in keeping with the finding of the present study of no significant association between students' health seeking behavior for menstrual problems and the age of students.

Women's lack of knowledge about expected changes in menstrual function across the life course often make it difficult for them to separate normal age-related changes in bleeding patterns from menstrual morbidity. Reproductive information programs that currently refer to menstruation in explanations of reproductive functions could easily incorporate more information about menstrual disorders and help break factors that prevent women from obtaining timely and appropriate care for menstrual dysfunction.
